# 

**Published:** 1849-10

**Authors:** 


PUBLISHED UNDER THE AUSPICES OF THE

American Society of Dental Surgeons.

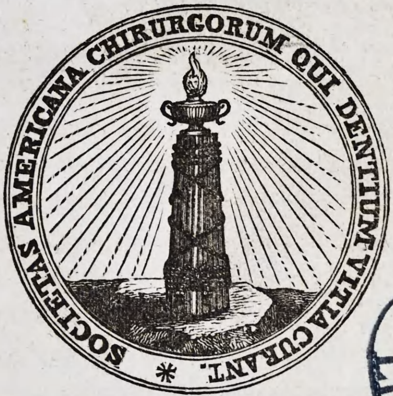

VOLUME X.—1849–50.

SURGEON GEN'L'S OFFICE
LIBRARY.
158315

EDITORS:

CHAPIN A. HARRIS, M. D., D. D. S.
AMOS WESTCOTT, M. D., D. D. S.
WILLIAM H. DWINELLE, M. D., D. D. S.

BALTIMORE:
ARMSTRONG & BERRY,
PHILADELPHIA: LINDSAY & BLAKISTON.
JOHN W. WOODS, PRINTER.

